# Exposure to Ketamine Anesthesia Affects Rat Impulsive Behavior

**DOI:** 10.3389/fnbeh.2016.00226

**Published:** 2016-11-24

**Authors:** António Melo, Hugo Leite-Almeida, Clara Ferreira, Nuno Sousa, José M. Pêgo

**Affiliations:** ^1^Life and Health Sciences Research Institute (ICVS), School of Medicine, University of MinhoBraga, Portugal; ^2^ICVS/3B’s - PT Government Associate LaboratoryBraga/Guimarães, Portugal

**Keywords:** ketamine, ketamine anesthesia, rat model, impulsive behavior, nucleus accumbens, striatum

## Abstract

**Introduction**: Ketamine is a general anesthetic (GA) that activates several neurotransmitter pathways in various part of the brain. The acute effects as GA are the most well-known and sought-after: to induce loss of responsiveness and to produce immobility during invasive procedures. However, there is a concern that repeated exposure might induce behavioral changes that could outlast their acute effect. Most research in this field describes how GA affects cognition and memory. Our work is to access if general anesthesia with ketamine can disrupt the motivational behavior trait, more specifically measuring impulsive behavior.

**Methods**: Aiming to evaluate the effects of exposure to repeat anesthetic procedures with ketamine in motivational behavior, we tested animals in a paradigm of impulsive behavior, the variable delay-to-signal (VDS). In addition, accumbal and striatal medium spiny neurons morphology was assessed.

**Results**: Our results demonstrated that previous exposure to ketamine deep-anesthesia affects inhibitory control (impulsive behavior). Specifically, ketamine exposed animals maintain a subnormal impulsive rate in the initial periods of the delays. However, in longer delays while control animals progressively refrain their premature unrewarded actions, ketamine-exposed animals show a different profile of response with higher premature unrewarded actions in the last seconds. Animals exposed to multiple ketamine anesthesia also failed to show an increase in premature unrewarded actions between the initial and final periods of 3 s delays. These behavioral alterations are paralleled by an increase in dendritic length of medium spiny neurons of the nucleus accumbens (NAc).

**Conclusions**: This demonstrates that ketamine anesthesia acutely affects impulsive behavior. Interestingly, it also opens up the prospect of using ketamine as an agent with the ability to modulate impulsivity trait.

## Introduction

General anesthetics (GA) are widely used in invasive surgeries and diagnostic procedures. Their application is considered to be safe, despite evidences suggesting post-surgery behavioral impairments. Following the pioneer work by Bedford (1955), several studies have investigated the relationship between surgical anesthetics and several clinical disturbances involving deteriorated memory and executive function (for review see Steinmetz et al., 2009). These functional alterations are now collectively called *postoperative cognitive dysfunction* (POCD). POCD is normally a transient phenomenon in the majority of patients, although it might gain a persistent character in aged individuals (Abildstrom et al., 2000; Selnes et al., 2008). POCD is not entirely understood and seems to be linked to a multitude of factors, such as the duration of surgery (Canet et al., 2003), specific procedures such as cardiac (Goto and Maekawa, 2014) and orthopedic surgery (Shi et al., 2014), immune response to surgery (Riedel et al., 2014) and to individual characteristics of each subject such as age or previous cognitive impairments (Moller et al., 1998; for review see also Krenk et al., 2010).

Ketamine is an intravenous GA, classified as a dissociative agent that acts as an antagonist in N-methyl-D-aspartate (NMDA) glutamate receptor (Maeng et al., 2008). There is evidence that ketamine interferes with dopamine release as well (Hancock and Stamford, 1999; Masuzawa et al., 2003) explaining its effect on executive function and motivation behavior. Others have argued that its effects mainly stem from its psychotomimetic properties and its ability to induce psychosis-like behavior in several animal models (Frohlich and Van Horn, 2014). Despite the potential behavioral secondary effects, ketamine remains a very versatile drug used in pain treatment, in general anesthesia and, more recently, in antidepressant therapy (DeWilde et al., 2015; Melo et al., 2015). Additionally, it is also used as a recreational drug with known addictive properties (Sun et al., 2014).

Despite the growing number of studies on this drug and associated effects, its impact on impulsive behavior remains largely unexplored. Impulsivity is a complex construct encompassing the domains of inhibitory control and decision-making (Dalley and Roiser, 2012). It is in most cases considered adaptive (trait impulsivity) but it can assume a disruptive character in several neuropsychiatric diseases as ADHD, substance dependence or obsessive-compulsive disorders. Impulsive behavior is often classified in impulsive action— compromised ability to inhibit a pre-potent behavior—and impulsive choices—preference for immediate though suboptimal over delayed but more compensating options (Evenden and Ryan, 1996).

Our objective is to study if repeated exposure to ketamine in the context of general anesthesia might induce changes in impulsivity using a paradigm that allows evaluating both impulsive action and delay intolerance.

## Materials and Methods

### Animals

Male Wistar rats (Charles-River Laboratories, Barcelona), weighing 300–400 g and aged 6 months, were housed (two per cage) under standard laboratory conditions (12 h light: 12 h dark cycle, at 22°C, relative humidity of 55%; free access to food and water). Sixteen animals were randomly assigned to two experimental groups—a control group (CONT) without anesthetic exposure and a group with ketamine exposure (KET). The dietary regimen was restricted to 1 h of food availability (19:00–20:00), 3 days preceding the initiation of the behavioral experiments. Body weight was controlled to ensure that it did not decrease below 85% of the initial value.

A different set of animals was assigned to perform behavioral evaluation after anesthetic exposure with the same protocol applied to the variable delay-to-signal (VDS) group. Animals performed the following behavioral tests: Elevated Plus Maze, Open Field, Forced Swim Test and Novel Object Recognition test.

Experiments involving the use of animals followed the guidelines of European Community Council Directive 2010/63/EU and were approved by the university ethical committee.

### Drugs

For the experimental design the following drug was used: Ketamine (50 mg/ml, Ketalar^®^, Pfizer, New York City, NY, USA) and as vehicle, saline (NaCl 0.9%).

### Anesthetic Procedure

**Figure 1:**
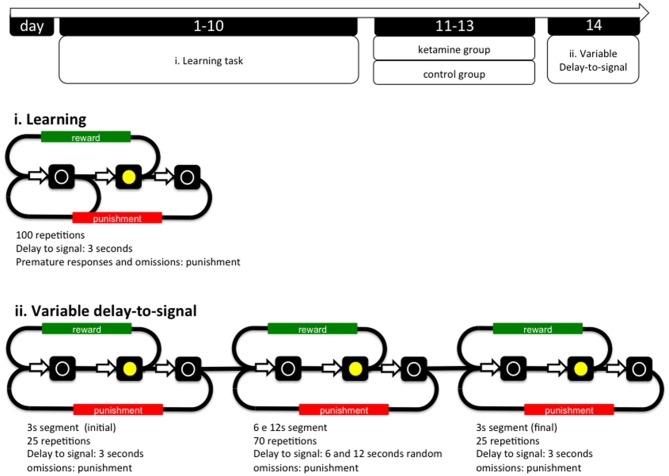
**General organization of the experiment and operational diagrams of the VDS and preceding training protocol.** The VDS consisted in two parts: the training protocol (10 sessions) and the VDS proper (1 session); while in the first the delay-to-signal was fixed (3 s) and pre-signal nose pokes were punished (TO), in the VDS proper, 2 blocks of 25 trials at 3 s delay were interposed by a block of 70 trials at 6 s and 12 s, pre-signal responses were registered but not punished with a TO. In both training and VDS the signal duration was set to a maximum of 60 s; the absence of a response within this period was registered as an omission. TO, timeout; VDS, variable delay-to-signal task.

The anesthetic procedure was performed through intra-peritoneal injections of ketamine according to the following scheme: an initial anesthetic dose of 100 mg/Kg of ketamine to induce anesthesia and then two subsequent injections of 50 mg/Kg. Anesthetized animals were placed over a warming pad with feedback control at 37°C in order to maintain body temperature. Anesthesia was considered adequate when animals lost the righting reflex and were irresponsive to tail pinch. The following dosage (50 mg/ml) was given when animals regain response to pressure stimuli in the tail. The anesthesia protocol was applied between the end of the training sessions and before the VDS test session (see below) in three GA procedures. Average time of anesthesia was 2.5 h. A washout period of at least 12 h was applied between the last anesthetic procedure and the initiation of the VDS protocol (see Figure 1).

#### Variable Delay to Signal (VDS)

The VDS task (Leite-Almeida et al., 2013) was performed in a 25 cm × 25 cm × 40 cm (W × L × H) operant chamber (OC; TSE Systems, Bad Homburg, Germany). The selection wall was slightly curved and presented five squared apertures (2.5 cm^2^), equally distributed and elevated 2 cm from the grid floor (during the VDS procedures only the middle aperture is available; see below). Similarly, on the opposite wall, an aperture (food access) led to a pellet dispenser. Each aperture contained a 3W lamp bulb and an infrared beam system to detect the activity of the animal. Four OCs were used simultaneously, each placed in soundproof boxes with individual electrical fans for ventilation and white noise production. Animals were habituated to the testing conditions once a day for 4 days (pm session). In the first 2 sessions, animals were placed in the OC for 15 min with all lights off, access to apertures 1–5 blocked by metallic caps and 10–15 sugared pellets (45 mg, Bioserv Inc., New Jersey, EUA) available in the reward-deliver aperture. In sessions 3–4, animals were placed in the OCs for 30 min, all lights were on and animals had free 2–3 pellets available in the central (#3) nose poke aperture 10–15 in the reward-deliver aperture. The protocol for VDS was initiated the following day and included two phases: (i) training sessions; and (ii) VDS test (see Figure 1).

**Figure 2:**
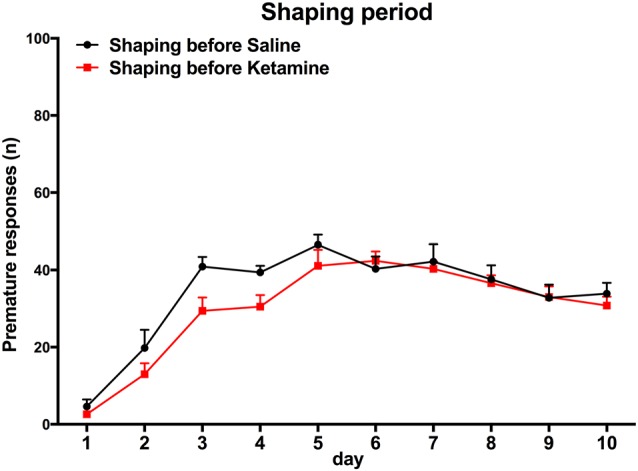
**Training session.** Animal performance in both groups does not differ significantly. Group assignment is performed after the end of the learning period, with animal randomly assigned to the groups and matched for performance in premature responses (PR).

(i) Training. Sessions were initiated by turning the house light on and delivering one sugared pellet in the reward-deliver aperture, the collection of which started an inter trial interval (ITI) of 3 s to allow the animal to ingest it. Trials then started, consisting of a 3 s period with only the house light on (delay period), followed by lightning of the response aperture #3 for 60 s (response period). Nose pokes in the delay period were punished with a timeout (TO) period in complete darkness (5 S) and in the response period (aperture #3 light on) were rewarded with the delivery of one pellet. These responses were respectively labeled as premature (PR) or correct responses. Collection of a food reward always triggered a 3 s ITI, before a new trial begun. Except for the TO periods, the house light was always on. Each session consisted of 100 trials or a maximum time of 30 min (whichever was reached first). Training sessions occurred twice per day, with a 5 h interval in between, for five consecutive days. The average number of PR of the overall group stabilized at ~30% in the last four sessions of training (see Figure 2).

(ii) Test. The VDS testing session occurred on a single day and consisted of 120 trials, similar to those previously described, with the exception of the delay, which was 3 s in the first and the last 25 trials, and, either 6 or 12 s in the middle of the 70 trials randomly attributed by the software leading to a 3 s (i)—6/12 s—3 s (f) configuration. Importantly, nose pokes were allowed during the delay period; these premature responses (PR) were registered and did not trigger TO or reward deliver.

VDS parameters analyzed are: PR/minute—number of PR per amount of delay; cumulative prematurity—is the average of PR accumulated along consecutive bins of 500 ms; latency to feed—average latency to collect the reward: response latency—amount of time between the light signal at #3 and response. Latencies are presented as averaged times, i.e., sum of response times divided by the number of trials at each block—3 s (i), 6 s, 12 s or 12 s.

#### Elevated Plus Maze

To assess anxiety-like behavior, animals were tested on the elevated-plus maze (MED-NIRPMNR; Med Associates, St Albans, VT, USA) as previously described (Pêgo et al., 2008). Animals were placed at the central hub facing the open-closed arm intersection and were allowed to explore the maze for 5 min. An arm entry was considered if the four paws were positioned within the arm. The test was filmed and the ratio between time in open arms and time in closed arms was subsequently quantified. Activity in the open arms was calculated as open arm entries percentage (entries into the open arms/total entries into all arms) and time spent in open arm percentage (time spent in the open arms/total time spent in all arms). The degree of anxiety was indirectly related to the time spent in the open arms and the number of open arm entries.

#### Open Field

To assess locomotor activity, rats were placed in an open-field apparatus (43.2 (length) cm × 43.2 (width) cm × 30.5 (height) cm, transparent acrylic walls and white floor, Med Associates) in a room illuminated by white light. Instant position was monitored over a period of 5 min by an array of two 16 beam infrared arrays. Total distance and average speed were used as a measure of locomotor activity.

#### Forced Swim Test

Learned helplessness was evaluated in the forced-swim test on the last day of exposure to uCMS. Twenty-four hours after a pre-test session (10 min), rats were placed in cylinders filled with water (25°C; depth 30 cm) for a period of 5 min. Test sessions were assessed using a camera connected to a video tracking system (Viewpoint, Lyon, France); the system automatically calculated immobility time and latency to immobility. Learned helplessness behavior was defined as an increase in time of immobility and a decrease in latency to immobility.

#### Novel Object Recognition

Recognition memory of the animals was assessed using an adapted version of the non-matching-to-sample learning task as previously described (Bevins and Besheer, 2006).

### Histological Procedures

After behavioral evaluation, five rats from each group were perfused transcardially with saline (NaCl 0.9%) under deep pentobarbital anesthesia. Brains were removed and kept in Golgi-Cox solution for 15 days and then transferred to a 30% sucrose solution for 5 days (Glaser and Van der Loos, 1981). Coronal sections (200 μm) were obtained using a vibratome and collected in 6% sucrose and blotted dry onto gelatin-coated microscope slides. They were alkalinized in 18.7% ammonia, developed in Dektol (Kodak, Rochester, NY, USA), fixed in Kodak Rapid Fix, dehydrated and xylene-cleared before coverslipping. Dendritic arborization was then analyzed in the nucleus accumbens (NAc) and Striatum (Str). Selected neurons had every branch of the dendritic tree reconstructed using a motorized microscope (Olympus BX51; Olympus, Tokyo, Japan) and Neurolucida v10 software (Microbrightfield, Williston, VT, USA) and three-dimensional analysis of the reconstructed neurons was performed using Neurolucida Explorer v10 software (Microbrightfield). For each animal, 20 neurons were reconstructed and measurements from individual neurons from each animal were averaged. The following dendritic morphology parameters were examined: dendritic length and the number of primary dendrites and dendritic branching points were compared across experimental groups. The observer who made the neuronal reconstruction was blind on the constitution of the groups.

### Statistical analysis

Data is presented as mean ± SEM and analyzed using two-way analysis of variance followed by a *post hoc* (Bonferroni) for multiple comparisons for analysis of the VDS results. Independent-samples *t*-test was used to test for the drug effect within each delay; behavioral results in the elevated plus maze, open field, forced swimming and novel object recognition test; and for analysis of neuronal dendritic length. The spearman rank correlation test was used to test correlation between different variables. Results were considered statistically significant if *p* < 0.05.

## Results

### Impulsivity

**Figure 3:**
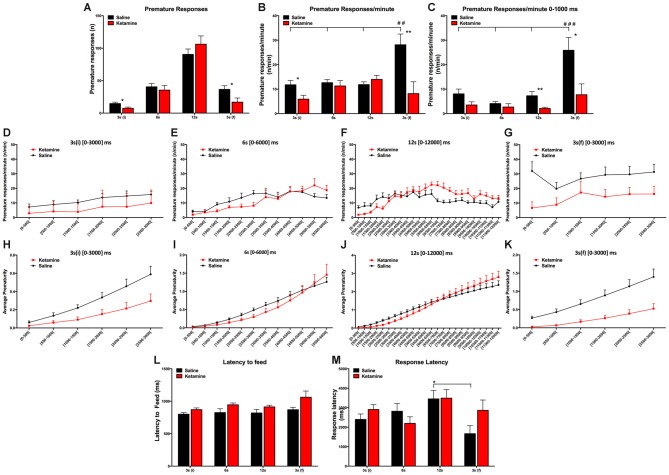
**VDS test results general anesthesia with ketamine had no effect in the absolute number of PR; however, the number of PR varied according to the delay (A)**. Normalized PR per minute of available delay (PR/m) varied according to the delay 3 s (i), 6 s, 12 s and 3 s (f). Decrease of PR/m in animals treated with ketamine compared to the control animals in both 3 s (i) and 3 s (f) delay periods **(B)**. Decrease of PR for the KET animals when compared with CONT animals in the 0–1000 ms period **(C)**. Absolute (**D–G**) and the accumulated (**H–K**) average prematurity/premature responses profile is presented in a segmented (500 ms periods) fashion for each delay segment 3 s **(i)**, 6 s, 12 s and 3 s **(f)**. No differences were observed in the latency to feed and no overall effect was detected across different delays **(L)**. No effects were observed in response latencies between experimental groups **(M)**. (*p < 0.05; **p < 0.01; ^##^*p* < 0.01; ^###^*p* < 0.001; *significances refer to differences between groups, ^#^significances refer to differences between different delays).

We tested the effect of repeated anesthetic exposure to ketamine in impulsivity behavior. General anesthesia with ketamine had no effect in the absolute number of PR (*F*_(1,112)_ = 0.6795 *P* = *ns*); however, the number of PR varied according to the delay (*F*_(3,112)_ = 59.74, *P* < 0.0001; Figure 3A). We also observed that the normalized PR per minute of available delay (PR/m) varied according to the delay 3 s (i), 6 s, 12 s and 3 s (f) (*F*_(3,112)_ = 4.087, *P* = 0.0085) and that it was significantly decreased for animals treated with ketamine compared to the control animals (*F*_(1,112)_ = 10.66, *P* = 0.0015) in both 3 s (i) and 3 s (f) delay periods (Figure 3B). Our results revealed that animals from both groups, CONT and KET, kept their PR/m rate relatively constant across the initial blocks, while for the last 3 s delay, we observed a difference in the IR: CONT animals showed a normal increase in PR/m, while KET were insensitive to the exposure to large delays (*F*_(1,112)_ = 6.931, *P* = 0.0015; Figure 3B).

To further explore the possibilities offered by VDS in the characterization of impulsivity and according to our previous studies (Leite-Almeida et al., 2013), we partitioned each delay block into 500 ms intervals (Figures 3D–K). This analysis confirmed our previous data showing a decrease of PR for the KET animals when compared with CONT animals (delay: *F*_(3,112)_ = 10.44, *P* < 0.0001; exposure to ketamine: *F*_(1,112)_ = 14.73, *P* = 0.0002; Figure 3C). In the 3 s (i) and 6 s delays, we observed no differences between groups. But in the 12 s delay, KET exposure decreased PR rate in the first part (up to 6000 ms) but not in the second part (6000–12,000 ms) of the delay, suggesting a heightened impulsive behavior (*p*_(*t* = 0.880, dF = 28)_ = 0.0075; Figure 3C). On the contrary, in the last delay period [3 s (f)] (*p*_(*t* = 2.654, dF = 28)_ = 0.0130), ketamine prevented an increase in PR that in normal conditions is observed after exposure to long delays (Figure 3C). These observations are not due to sedative or motivational effect at the selected doses as no differences were observed in the latency to feed and no overall effect was detected across different delays (*F*_(3,112)_ = 2.504, *P* = 0.0629; Figure 3L). No effects were observed in response latencies between experimental groups (drug*delay: *F*_(3,112)_ = 1.886, *P* = *ns*; Figure 3M).

### Other Behavioral Evaluations

**Figure 4:**
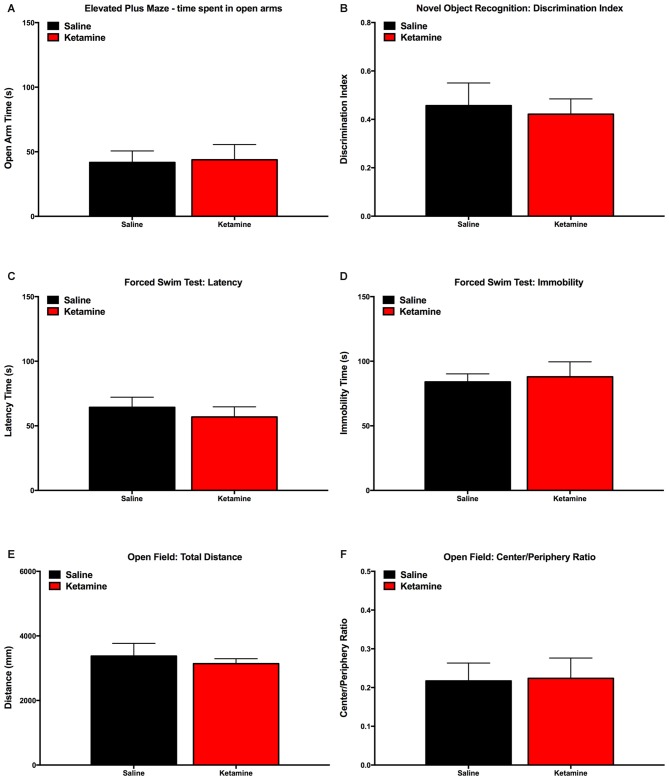
**Behavior evaluation performed to validate the model of anesthesia exposure:** animals exposed to ketamine anesthesia performed similarly to control animals in the Elevated Plus Maze **(A)**, Novel Object Recognition Test **(B)**, Open Field **(C,D)**, Forced Swimming Test **(E,F)**.

In order to rule out the effect of ketamine anesthesia behavioral dimensions that could interact with VDS results, we performed evaluations in a different group of animals not assigned to perform the VDS paradigm. Results show that animals exposed to ketamine anesthesia have similar performance to control group concerning Elevated Plus Maze (Figure 4A), Novel Object Recognition Test (Figure 4B), Forced Swimming test (Figures 4C,D) and Open field (Figures 4E,F).

### Neuronal Morphology

**Figure 5:**
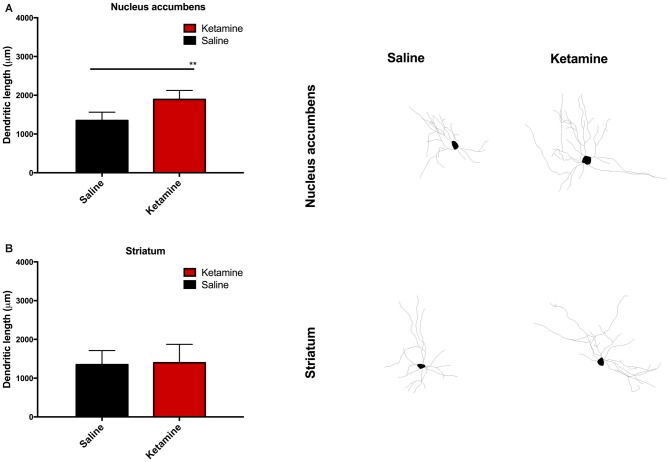
**Neuronal dendritic reconstruction in the nucleus accumbens (NAc).** Increase in dendritic length in animals exposed to ketamine anesthesia **(A)** and no difference in the striatum analysis **(B)**. (**p < 0.01).

We performed neuronal dendritic reconstruction. The analysis of dendritic branching in the NAc showed a significant increase in dendritic length in animals exposed to ketamine anesthesia *p*_(*t* = 3.880, dF = 28)_ = 0.0047 (Figure 5A) and no difference in the striatum analysis (Figure 5B). No differences were found concerning dendritic spine analysis.

### Correlation of VDS Performance With Neuronal Morphology

**Figure 6:**
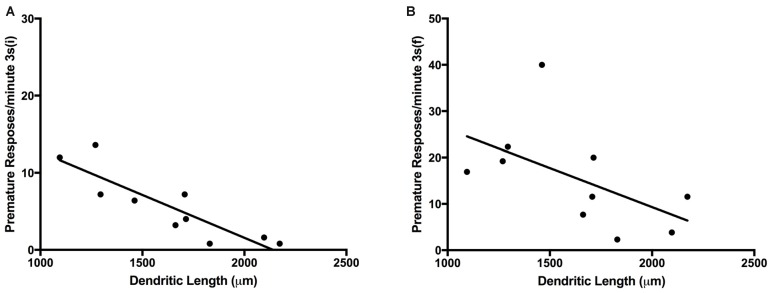
**Plot graph;** values of ratio of PR/minute in the 3 s initial period **(A)**; values of ratio of PR/minute in the 3 s final period **(B)**; (PR, premature responses).

We found a significant negative correlation between NAc neuronal dendritic length and the number of absolute PR in the initial 3 s delay (*R* = −0.67, *P* = 0.039) and final 3 s delay PR 3 s (i) (*R* = −0.66, *P* = 0.041). Concerning the ratio of PR/minute we also found a negative correlation between the values in the initial 3 s period and dendritic length of NAc neurons (*R* = −0.740, *p* = 0.001) (Figures 6A,B; Table 1).

**Table 1:** Correlation table (Pearson *r*^2^ values; *P* values; *p < 0.05).

	Dendritic length nucleus accumbens	
	*R*^2^	*p*
Premature responses/minute 3 s (i)	−0.89*	0.001093474
Premature responses/minute 6 s	−0.27	0.448276565
Premature responses/minute 12 s	0.09	0.811282518
Premature responses/minute 3 s (f)	−0.57	0.093003748

## Discussion

In this work, we investigated the existence of possible alterations in executive function induced by consecutive ketamine anesthetic administration using impulsivity as surrogate readout as assessed by the VDS. We observed that KET-multiple exposure abolished the increase in the rate of PR (PR/minute) that is generally observed in the last block [3 s (f)] after the long-delay trials (6 and 12 s; Leite-Almeida et al., 2013). This effect was preserved in control animals. Curiously, over the course of long 6 s and 12 s delays, KET animals increased their PR above normal levels. It is important to notice that we found no differences in the latencies to feed or changes in animal weight, indicating that both KET-exposed animals and controls were equally motivated during the performance of the test. Additionally, we have analyzed the neuronal dendritic architecture in the NAc and striatum; our analysis revealed an increased average dendritic length in the first. Also, we observed increased impulsivity at the 3 s (i) and 3 s (f) blocks were associated with longer dendritic length in the NAc medium spiny neurons.

The VDS, which was developed in our lab, was designed and validated to evaluate impulsive behavior in a rat model (Leite-Almeida et al., 2013). In this paradigm, delay intolerance manifests as a robust increase of PR in the 3 s (f) segment when compared to the 3 s (i), i.e., after exposure to large 6 s and 12 s delays. This increment in the rate of impulsive responses as well as the rate of impulsive responses in the later periods of the 12 s segment has been shown to correlate with discounting steepness in the delay discounting (DD) paradigm (Evenden and Ryan, 1996). Additionally, impulsive responses in the training phase reflect action impulsivity; the construct at this stage is similar to that of the 5-choice serial reaction time task (5-CSRTT; Carli et al., 1983).

The KET group displayed a smaller PR/min in the 3 s (i) delay, even when the analysis was confined to the first 1000 ms, a parameter that is closely associated with impulsive action (Leite-Almeida et al., 2013). In previous works, using Sprague Dawley (Nemeth et al., 2010) and Lister Hooded (Smith et al., 2011) rats under the influence of acute sub-anesthetic doses of KET showed no difference in impulsive responses in the 5-CSRTT. Similarly, Nikiforuk and Popik (2014) observed that exposure to both single or repeated sub-anesthetic doses of ketamine produced no differences in the baseline performance of any of the 5-CSRTT performance measures, such as correct/incorrect responses, omissions, accuracy and perseverant responding). However, in both experimental conditions an increase in omissions was detected without alterations in motivational aspects of the response. On the contrary, Oliver and coworkers revealed that acute administration of sub-anesthetic doses of ketamine increased the CD1 mice impulsive behavior, when performing the 5-CSRTT (Oliver et al., 2009). Indeed, several studies reported that NMDA antagonists (other than ketamine) increase impulsivity in the 5-CSRTT. That is the case of acute treatment with MK-801 or phencyclidine (Paine and Carlezon, 2009; Thomson et al., 2011; Barnes et al., 2012). Similarly, our group has shown that an acute challenge with MK-801 is able to increase all measures of impulsive behavior in the VDS (Leite-Almeida et al., 2013). On the other hand, chronic treatment may display a different behavioral pattern, either reduced PR during a 24-h drug withdrawal (Paine and Carlezon, 2009) or had no effect (Thomson et al., 2011; Barnes et al., 2012). Until now, most research seems to show a tendency of NMDA antagonists to increase impulsive behavior after an acute exposure, while on sub-chronic and chronic regimens the response seems to be towards a decrease in impulsive response particularly if the test is made during the withdrawn period. Our study shows a more complex picture. In line with the above studies, we observed a decrease in basal impulsive response. However, when analyzing the patterns of response along the delay period in intervals of 500 ms, it was evident that KET animals increased their PR/min towards the end of the delay overcoming the CONT group. In other words, while CONT animals, after some premature unrewarded responses, were able to correct their behavior, KET animals failed to adjust and steadily increase their prematurity rate, suggesting failures in their inhibitory control. In addition, KET animals failed to show a robust increase in the PR/min between [3 s (i)] and [3 s (f)], which is normally observed after the exposure to large delays (Leite-Almeida et al., 2013). The PR/min in the [3 s (f)] is strongly correlated with the DD behavior, i.e., higher PR/min is associated to a preference for immediate (but smaller) choices in the DD (Leite-Almeida et al., 2013). Our findings suggest that ketamine disrupted a response correlated with performance in DD. These findings are supported by data from studies that show changes in DD performance after administration of ketamine and other NMDA antagonists such as memantine and MK-801 (Floresco et al., 2008; Cottone et al., 2013; Yates et al., 2015). Specifically, Yates et al. (2015) observed that ketamine decreased the sensitivity to reinforcer amount in DD, while Cottone showed that ketamine increased the choice for small immediate reward (Cottone et al., 2013) and Floresco reported that ketamine induced a decrease in tolerance for delayed rewards (Floresco et al., 2008).

No significant differences were observed in response and feed latencies which is concordant with the work of Nelson et al. (2002) where repeated ketamine administration was shown to have no impact on animals’ capacity to perform the sustained attention task.

Food availability is also a concern and a limitation in operant behavior protocols. Research animals are food restricted in order to get the motivation for performing the task. The specific feeding regimen was applied according to the previously published protocol of the VDS paradigm (Leite-Almeida et al., 2013) and following the recommendations for daily food intake previously reported in other operant protocols (Bari et al., 2008). However, binge access to food might also promote the development of a higher impulsive trait (Vanderschuren and Ahmed, 2013).

VDS results were also not affected by emotional, locomotory or cognitive altered behavior as there were no differences between experimental and control group in these behavioral tests.

Chambers and Potenza (2003) proposed two main circuits to control impulsive response. A primary circuit consisting of parallel loops of neuronal projections from the prefrontal cortex, to the ventral Str (including the NAc), to the thalamus and then back to the cortex and a second circuit would supply the primary with autonomic, affective, motor and memory information necessary for a proper shaping of the output. The role of NAc in impulsive behavior was first shown by Cardinal et al. (2001), who described that bilateral lesion of the NAc but not of the anterior cingulate cortex or medial prefrontal cortex, resulted in more impulsive choices in delayed reinforcement choice task. In the DD, NAc lesioned rats were less impulsive, but only when the delay was changed between sessions, suggesting that NAc lesions impaired learning or adaptation to changes in delay reinforcement but did not affect tolerance to delays (Acheson et al., 2006). On the contrary, da Costa Araújo et al. (2009) showed that NAc lesioned rodents had changes in inter-temporal choice behavior preferring immediate over delayed reinforcement in an adjusting-delay approach. In our work, KET exposure increased dendritic arborization in the NAc medium spiny neurons but not in the striatum. Drugs of abuse with known effect in impulsive behavior, such as amphetamine and cocaine, are known to induce changes in neuronal architecture in the NAc (Robinson and Kolb, 2004). Ketamine is a GA that is also used as a drug of abuse. Despite its relatively short half-life, some of the effects of ketamine are known to be long-lived, namely through the activation of signaling pathways following the acute blockage of NMDA receptors and that are responsible for the neuronal changes namely synaptic and dendritic modeling. Exposure to a single dose of ketamine is known to increase the levels of activity regulated cytoskeletal protein (Arc), glutamate AMPA receptor-1 (GluR1), postsynaptic density protein-95 (PSD95) and synapsin I, all of them of importance in dendritic and synaptic remodeling (Li et al., 2010). Ketamine also has been shown to regulate the levels of neurotrophic factors such as brain derived neurotrophic factor (BDNF), which has been related with processes such as neurogenesis and synaptic protein expression (Kovalchuk et al., 2002). Ketamine also affects the regulation of signaling pathways such as mammalian target of rapamicin (mTOR), is a critical mediator of protein synthesis (Ma and Blenis, 2009) including dendritic/synaptic proteins (Liu-Yesucevitz et al., 2011). Indeed, the inactivation of mTOR pathway blocked the antidepressant effect of ketamine (Li et al., 2010). Interestingly, Sabino et al. (2013) showed that mTOR activation is needed to achieve a reduction of alcohol consumption in alcohol-preferring rats, showing that this pathway is also implicated in the build-up of other behaviors such as addiction. Our findings of changes in neuronal architecture after exposure to ketamine supports the known effect in synaptic protein expression factors and regulation of protein synthesis signaling pathways such as the aforementioned mTOR pathway. The synthesis of synaptic proteins important for neuroplasticity is one of the potentially critical steps for ketamine action (Duman et al., 2012). Our findings on dendritic organization largely reflect these observations.

In summary, this study demonstrated that a multiple exposure to ketamine anesthesia affects impulsive behavior. We demonstrated that this abnormal inhibitory control is associated with an increase in neuronal dendritic length of the NAc. In human patients exposed to anesthetic procedures, the postoperative cognitive deficits, manifested by their inability to engage successfully tasks at home or work, have also been reported, though impulsive behavior has not been specifically measured. We cannot therefore exclude the possibility that alterations in impulsive behavior might also influence postoperative period impairments.

## Author Contributions

AM, HL-A, CF, NS and JMP are all substantial contributors to the conception, design, acquisition, analysis and interpretation of the experimental work; all took part in revising it and approval of the version to be published.

## Funding

This study was funded by Fundação para a Ciência e Tecnologia (FCT) grant SFRH/SINTD/60126/2009.

## Conflict of Interest Statement

The authors declare that the research was conducted in the absence of any commercial or financial relationships that could be construed as a potential conflict of interest.
